# Redeposition-free inductively-coupled plasma etching of lithium niobate for integrated photonics

**DOI:** 10.1515/nanoph-2022-0676

**Published:** 2023-01-16

**Authors:** Fabian Kaufmann, Giovanni Finco, Andreas Maeder, Rachel Grange

**Affiliations:** ETH Zurich, Department of Physics, Institute for Quantum Electronics, Optical Nanomaterial Group, Zurich, Switzerland

**Keywords:** argon etching, inductively-coupled plasma etching, integrated photonics, redeposition, thin-film lithium niobate

## Abstract

Lithium niobate on insulator is being established as a versatile platform for a new generation of photonic integrated devices. Extensive progress has been made in recent years to improve the fabrication of integrated optical circuits from a research platform towards wafer-scale fabrication in commercial foundries, and optical losses have reached remarkably low values approaching material limits. In this context, argon etching of lithium niobate waveguides has been shown to provide the best optical quality, yet the process is still challenging to optimise due to its physical nature. Namely, the micro-masking effects introduced by the material redeposition and a close to one etch mask selectivity for deep etches. We present a workflow to identify the parameter set offering the best etching results independent of the plasma system being used. We show how to reach the redeposition-free regime and propose three methods to achieve redeposition-free lithium niobate etching with good quality sidewalls without need of wet chemistry for cleaning.

## 1 Introduction

Since the advent of ion-slicing techniques [1, 2], thin-film lithium niobate on insulator (LNOI) photonics has seen rapid development due to the outstanding performance in terms of losses [3], transparency window [4–7], electro-optic bandwidth [8–14], scalability, and potential for integration. High electro- and thermo-optic coefficients [15] and its anisotropy make LNOI a versatile platform for a new generation of photonic integrated devices [16–20]. Additionally, the non-centrosymmetric structure of the crystal enables engineering of second order nonlinearities for both classical [21–24] and quantum applications [25–28].

In this paper, we characterise the etching of LNOI by means of argon sputtering in inductively-coupled plasma (ICP) reactive-ion etching system. Patterning of lithium niobate with dry etching techniques is known to be challenging due to the by-products created during the etch. Argon (Ar) based processes have been shown to provide the smoothest sidewalls [29–31] and allow the manufacturing of photonic integrated circuits of the best optical quality, with reported propagation losses as low as 2.7 dB/m in multimode structures [3, 9, 32] operating in the telecom C-band. Nevertheless, being of purely physical nature, Ar sputtering does introduce several complications, the most prominent being the impossibility of achieving vertical sidewalls and the redeposition of non-volatile etched material accumulating on the sidewalls. Ar mixtures with fluorine and chlorine gases have also been investigated [10, 33], [34], [35], [36] and although they allow achieving more vertical sidewalls, by-products introduce complications in terms of contamination and roughness, thus increasing optical losses. It has been shown that, in the context of integrated optical circuits, mode properties (especially the transverse-electric mode, TE) and device density do not suffer dramatically from the angled walls [29], but pose a strict limitation on the realisation of phase-controlling elements (i.e., photonic crystals, metasurfaces, etc.) [37–39]. Various methods have been proposed and shown to be successful in removing redeposition from the sidewalls by means of wet chemistry [9, 40], [41], [42]. However, to the best of our knowledge, no study has been conducted on the possibility of achieving redeposition-free dry etching with Ar sputtering. As such, we study the influence of ICP parameters on the resulting etched features with particular emphasis on redeposition removal, and we successfully identify ways of achieving this directly during the dry etching process. Optical losses in lithium niobate circuits are limited by scattering from the sidewall roughness, which is introduced by micro-masking effects [32] in part due to material redepositing on the etched walls. In order to fully benefit from the polishing character of Ar sputtering [29], redeposition removal during the dry etching would enable smoother sidewalls and lower optical losses.

We propose three methods to achieve redeposition-free lithium niobate dry etching and provide a workflow to identify the parameter set independent of the ICP-system being used. The most straightforward implementation of the process is based on a careful balance of ICP parameters, especially DC bias (acceleration voltage) and chamber pressure (depending on the final etch depth to be achieved). We observe that proximity of additional features to the target structure aids redeposition removal due to ion deflection between neighbouring walls and thus effectively increasing the lateral component of the etch. Lastly, we find that due to the dynamics of the redeposition process, its removal can be favoured by tailoring the etching mask accurately towards a trapezoidal shape.

We will comment on these points and highlight their advantages and drawbacks in terms of feasibility and practicality, but also in terms of influence on machine conditions and suitability from a research rather than industrial context.

## 2 Methods

### 2.1 Wafer preparation

A full 4-inch LNOI wafer with a 400 nm x-cut film and 2 μm SiO_x_ insulation layer (from NANOLN) is exposed in a 100 kV electron beam lithography (EBL) system (Vistex EBPG 5200+, Raith). In a first step, positive EBL markers for the next exposure are fabricated using a double layer lift-off process. The metal for the markers was evaporated in an electron beam evaporator with a total thickness of 85 nm (5 nm Cr, 80 nm Pt), yielding a total of 52 10 × 10 mm^2^ samples, each with a set of alignment markers. Each sample has an effective area of 5 × 5 mm^2^ into which the design can be placed. After lift-off, a hydrogen silsesquioxane (HSQ)-based flowable oxide (FOX16, from DuPont/Dow Corning) is spun as negative EBL resist with a thickness of about 500 nm on the full wafer, which is then exposed with the photonic structures using a bulk-sleeve approach to minimise write-time. The sleeve is 150 nm thick and overlaps 50 nm with the bulk of the structure. To minimise the impact of beam fluctuations [43], the sleeve is written using four passes at a dose of 1900 μC/cm^2^, a beam current of 8 nA, resolution of 1 nm and beam step size of 5 nm. The bulk is written with a dose of 7600 μC/cm^2^, a beam current of 100 nA, a resolution of 1 nm and beam step size of 25 nm. Developing is done for 5 min in a 1:3 AZ351B:DI-H_2_O solution. The developed resist has a sidewall angle of 85° and shows minimal sidewall roughness and high resolution expected from HSQ and FOX with gaps of 100 nm still well resolved. The wafer is then diced into a total of 52 10 × 10 mm^2^ samples patterned with both test structures and optical resonators of varying sizes. Importantly, samples are patterned in a way such that the extraordinary (Z) crystal axis is orthogonal to the straight structures in order to study the influence of crystal orientation on the etch.

### 2.2 ICP etching

The samples are wax-glued to a 4-inch silicon carrier wafer and etched in an ICP (Plasma Pro 100, Oxford Instruments). Before the start of the experiment, the machine is cleaned manually using Scotch Brite with DI water and wiped down using IPA. An O_2_ burn of 4 h, using optical emission spectroscopy (OES) to measure the plasma emission lines, ensures that no residual particles from the cleaning remain in the chamber. We would like to note that during the full time of the experiment only Cl_2_, SF_6_, O_2,_ and Ar gasses are allowed to be used in the machine as we found that especially CHF_3_ can have a strong impact on machine performance and reproducibility of the etching process and leads to strong contamination of the chamber.

To ensure a consistent etch depth between 200 nm and 250 nm (depending on the run), an interferometric endpointing system (Horiba) with a wavelength of 670.1 nm is used. This approach allows us to not have to normalise to etch depth while comparing results. Prior to the experiment we found that the power of the plasma does only have a minor impact on the wall redeposition and selectivity, if DC bias and pressure are kept constant. Thus we can vary the ICP power such that the final etch rate of the process is within an acceptable range for the enpointing system. The samples are aligned in such a way that the polarisation of the beam follows the ordinary axis of the lithium niobate crystal. Before each test run the machine is cleaned in a SF_6_/O_2_ plus O_2_ plasma to remove residuals left by other processes and to reset the machine to a known state. After etching, the machine is cleaned in the same manner to remove oxide layers on the walls of the chamber, which can build up during the tests.

### 2.3 Characterisation and process description

The samples are inspected by scanning electron microscopy (SEM, ULTRA 55 plus, Zeiss), atomic force microscopy (AFM, Dimension FastScan, Bruker) and reflectometry. Focused ion beam milling (FIB, Helios 5 UX, ThermoFisher Scientific) is used to investigate the cross-section of the devices. We measure sidewall angle, etch depth and trench depth as well as the redeposition on both the +Z and −Z side of the structure. Optical measurements of on-chip resonators are performed to determine the optical quality provided by each parameter set of the etch.

**Figure 1:**
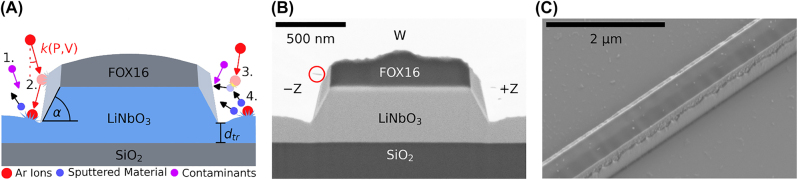
Overview of the processes taking place during etching. (A) Schematic of the etching process with details about the different mechanisms taking place. From left to right: (1.) contaminants intrude the process and change stoichiometry and nature of the redeposition; (2.) a trench appears at the bottom of the etched features due to the physical nature of the etch and thus ions impinging the substrate at grazing angles; (3. and 4.) Ar ions bombard the target and sputter material off it with etched specimens either escaping the substrate or getting redeposited on the features walls due to, e.g. re-collision with incoming ions. Definition of sidewall angle *α* and trench depth *d*_tr_ used in this work is shown as well. (B) FIB cross-section of a 1 μm wide structure covered in tungsten to protect against beam damage with voids due to non-uniform coverage, circled in red. −Z and +Z indicate the crystal faces. (C) SEM image of a 500 nm wide etched feature with mask still on top. The redeposition distribution along the +Z sidewall is clearly visible.

Figure 1(A) illustrates the physical mechanisms of Ar etching of lithium niobate. Ar ions are directed towards the substrate with trajectory and energy dictated by the process parameters, in particular pressure *P* (rate of collisions between ions) and DC bias *V*. The *k*-vector of the ions trajectory is a function of *P* and *V* and given by *k*(*P*, *V*).

Contaminants sputtered from the chamber walls, carrier substrate, wafer clamp etc. by the ion bombardment can be present as well and can contribute to micro-masking (Figure 1(A) 1.). During the process, a trench can appear due to deflected ions from the sidewall adding to the local etch rate close to the structure (Figure 1(A) 2.). Nonvolatile by-products (sputtered specimen) redeposit on the sidewalls of the mask and etched feature lead to the formation of redeposited lithium niobate and micro-masking (Figure 1(A) 3. and 4.). The latter can be measured by means of FIB analysis, as the redeposition is of different crystalline phase and composition than the target crystalline material [44], hence it can be identified with electron microscopy due to contrast. We measure the redeposition in terms of area as seen in Figure 1(A) and (B) (light grey area). Figure 1(B) shows a tilt-corrected FIB cross-section of a 1 μm wide structure covered in 3 nm of Cr to help with charge compensation and a protective layer of tungsten (W) deposited during FIB to protect from beam damage during cutting. The black spots in the tungsten, circled in red, are voids formed during the deposition due to non-uniform coverage. Figure 1(C) shows a rotated and tilted SEM image of an etched structure before cross-sectioning with the redeposition clearly visible on the sidewalls as well as flakes of material on the remaining thin-film. The concentration of the flakes increases close to etched structures and vanishes with increasing ICP power (plasma density). The surface root mean square height (Sq) value of an un-etched sample is measured to be 0.31 nm on a 1 μm^2^ area via AFM.

## 3 Results

### 3.1 Influence of the DC bias

**Figure 2:**
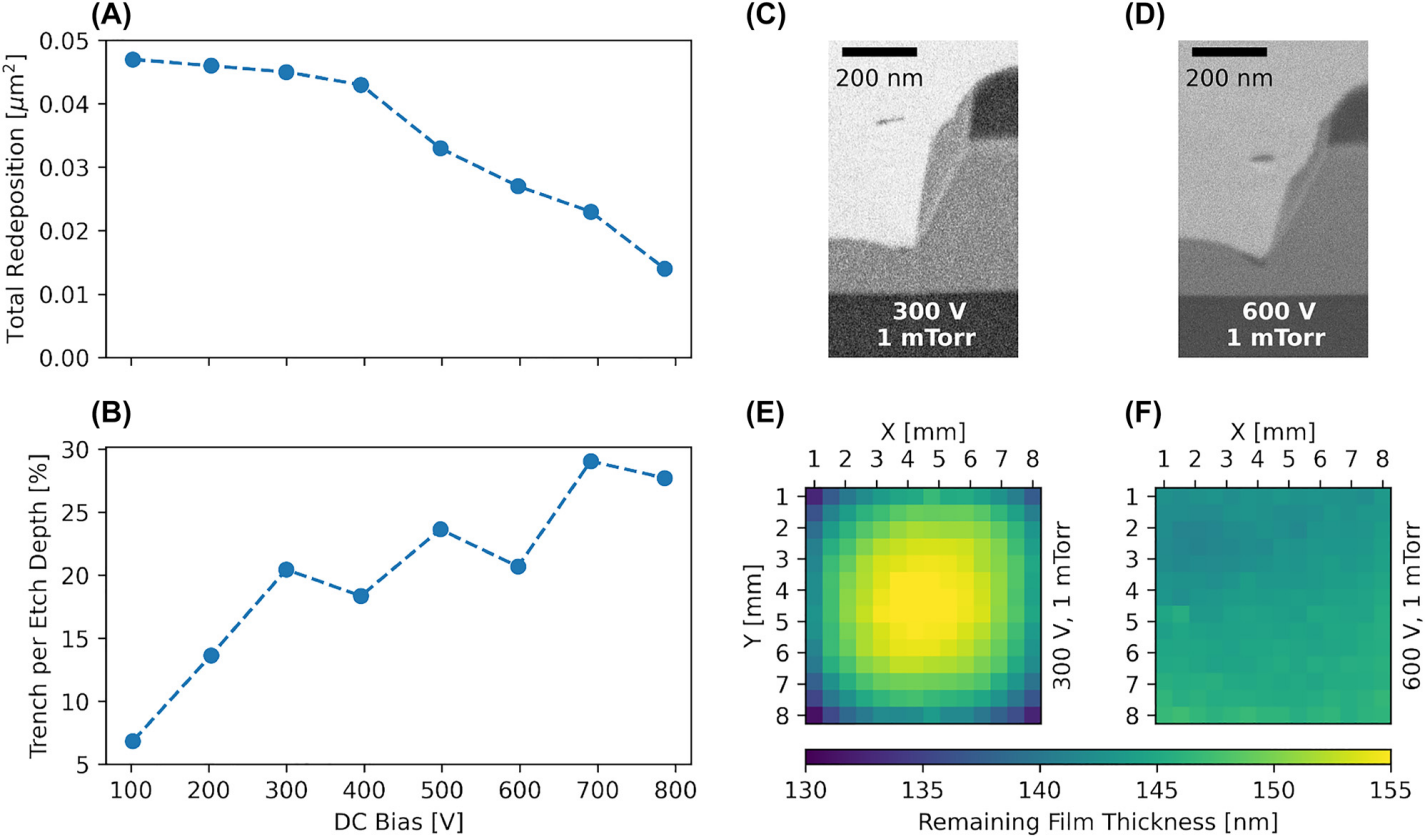
Influence of the DC bias. (A–B) Evolution of total redeposition area and trench depth per etch depth versus DC bias. (C–D) One-sided FIB cross-sections for samples etched with 300 V and 600 V, respectively. (E–F) Reflectometer measurements of the remaining thin-film for the same samples to illustrate the influence of DC bias on sample bow.

Initial tests show that the power of the plasma does not have a significant impact on the redeposition nor the selectivity if the DC bias and pressure are kept constant. This allows us to sweep the DC bias while simultaneously adapt the plasma power to keep the etch rate in a regime where the machine endpointing software can still trigger reliably at the desired etch depth. We vary the DC bias from 100 V to 800 V while keeping Ar flow at 10 sccm, chamber pressure at 1 mTorr and sample temperature at 20 °C to determine the trend of redeposition on the sidewalls. Figure 2(A) and (B) show the decrease of the total redeposition after etching as well as the increase of the trenching per etch depth with increasing DC bias. The increase in trench depth by a factor of five is explained by high energy ions impinging at an angle being deflected closer towards the foot of the etched structure, thus leading to a locally higher etch rate. Figure 2(C) and (D) display the one-sided FIB cross-section for two samples etched at 300 V and 600 V, respectively. It shows how an increased DC bias does provide a way of progressively removing material from the sidewalls as we measured a total redeposition area almost four times smaller for an etch performed at 800 V compared to the initial value of 100 V. This is explained with the impinging ions having enough energy to compensate for the redeposition rate, despite having less area of attack as seen from the point of view of an incoming ion compared to the point of view of the sputtered material (etch rate vs. redeposition rate). The data tends to a redeposition-free etch for biases above 1 kV, yet this is not a readily available configuration. We would also like to note that DC biases of 700 V and 800 V are hard to maintain consistently over the full time of the etch and 900 V is not achievable in our system. This effect is reproducible with different samples and is just as much about what happens on the sample or in the plasma, rather than the machine destabilising as such, and is open to interpretation. Moreover, the relative depth of the etched trenches increases as well by a factor of five, increasing the effective etch depth and optical mode confinement.

We observe no significant effect on the selectivity, nor on the sidewall angle, from increasing the DC bias above 200 V as is expected for a purely physical process at constant pressure. We report a selectivity (material etched vs. mask etched) close to one and an angle of 60–63° for both +Z and –Z sides of the crystal, as is reported in various sources [17, 29, 41]. For 100 V we observe a selectivity below one which is explained by the fact that both mask and material have a different sputter threshold (the one for LN being higher than for FOX16 used as a mask here). Using 50 V results in barely any LN being etched compared to the mask. Lastly, Figure 2(E) and (F) show reflectometer measurements in the visible range using a Halogen lamp for samples etched at 300 V and 600 V, where the effect of plasma focusing is visible. At low DC biases, tip effects at the samples edges cause a distortion of the plasma that result in a bow of the film thickness across the sample. The focusing of the plasma changes with increasing DC bias and the resulting bow of the etched sample changes from concave to flat to convex. We find that for 300 V the sample shows a thickness variation of 20 nm over an area of 8 × 8 mm^2^, whereas for 600 V the sample shows a thickness variation below 5 nm. The DC bias has no effect on surface roughness as the Sq values for all samples etched with more than 200 V lie around 0.08 nm measured on a 1 μm^2^ area, four times less than before etching. This shows the potential for very smooth and high quality sidewalls achievable by Ar etching provided the correct set of etching parameters to overcome the redeposition rate is used.

### 3.2 Influence of the chamber pressure

**Figure 3:**
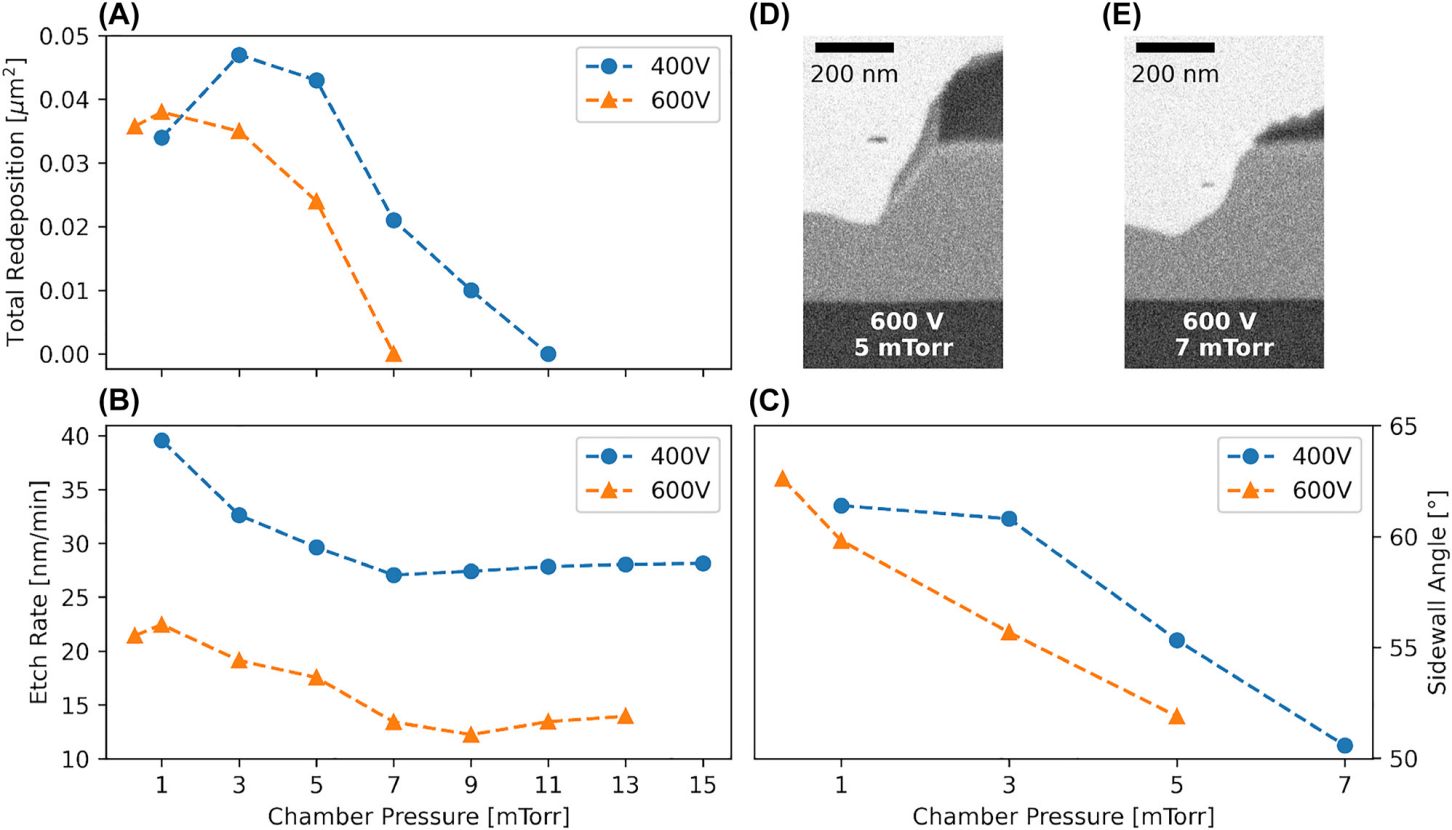
Influence of chamber pressure. (A) Evolution of total redeposition with respect to chamber pressure. Values for which the redeposition reaches zero are omitted for readability of the plot. (B) Etch rate and (C) sidewall angle versus chamber pressure. All three decrease with increasing chamber pressure. Note that the offset between the two etch rate curves at 400 V and 600 V is due to a different ICP power used for both cases. ICP power used for the 400 V etch is 600 W and for the 600 V etch 300 W. (D–E) One-sided FIB cross-sections for samples etched at 5 and 7 mTorr with 600 V DC bias.

To increase the lateral component of the etching and further bring the local etch rate near the structure closer to the redeposition rate, we sweep the pressure from 1 mTorr to 15 mTorr with a DC bias of 400 V and ICP power of 600 W and 0.3 mTorr to 13 mTorr with a DC bias of 600 V and ICP power of 300 W. Selectivity stays constant within measurement error until lateral etching starts to dominate at around 9 mTorr (400 V) and 7 mTorr (600 V), and the 1 μm wide mask starts degrading. The trench-to-etch-depth ratio is roughly 1:5 throughout the full pressure sweep, similar to the value reported above. Figure 3(A) shows the trend of the total redeposition, which starts to completely vanish after 7 mTorr, but with significant damage to the structure due to the jagged nature of the redeposition (see Figure 1(C) for a picture of the sidewall redeposition). The contribution of the redeposition acting as additional masking material leads to a slanted shape of the combined mask of FOX16 and redeposition. This leads to a second, steeper sidewall angle after significant enough buildup of redeposition has taken place (see Figure 3(D) and (E)). Figure 3(B) shows a decrease in etch rate of the material with increasing pressure, with a minimum value of 27 nm/min and 12 nm/min for the two DC bias voltages. This effect has been reported in [31] and is attributed to the etched material not being able to reliably escape the surface anymore and thus depositing directly on top of it, reducing the overall measured etch rate. It is also worth noting that there is a threshold at 7 mTorr and 9 mTorr (for 400 V and 600 V) after which the etch rate starts to increase again. We argue that in this regime the lateral component of the ions *k*-vectors starts to dominate the etching process and the etched material has enough energy to escape. We measure the sidewall angle where a single angle is still well defined (as seen in Figure 3(D)) and it decreases measurably for both the –Z and +Z side of the structure as is shown in Figure 3(C). We do not measure a significant effect on the sample bow after etching for different chamber pressures. Comparing the redeposition in Figure 2(C) with Figure 3(D), one can see a contrast difference, suggesting a change in composition towards lighter elements of the redeposition depending on the chamber pressure when approaching the redeposition free regime. This is either due to sputtered contamination from the chamber, hinting at how clean and well maintained it has to be, or sputtered material from the Si carrier wafer or mask. Here, the mask is the most probable source. The exact composition needs to be investigated further using, e.g. atom probe tomography (APT), as an energy dispersive X-ray (EDX) composition analysis on such small structures does not yield meaningful results and Li is too light to be detected. Measured surface roughnesses Sq for 3 mTorr, 5 mTorr, and 7 mTorr are 0.10 nm, 0.12 nm, and 0.15 nm, respectively, showing a degradation of the surface. Although still better by a factor of two compared to the un-etched surface, lower pressures are favourable.

Since the pressure required for a redeposition-free etching regime is lower for 600 V (5–7 mTorr) than for 400 V (around 11 mTorr), we select 600 V as our preferred DC bias voltage. As noted above, at this value, the provided samples are flat and have negligible plasma-induced thickness variation.

### 3.3 Aspect ratio dependent etching

**Figure 4:**
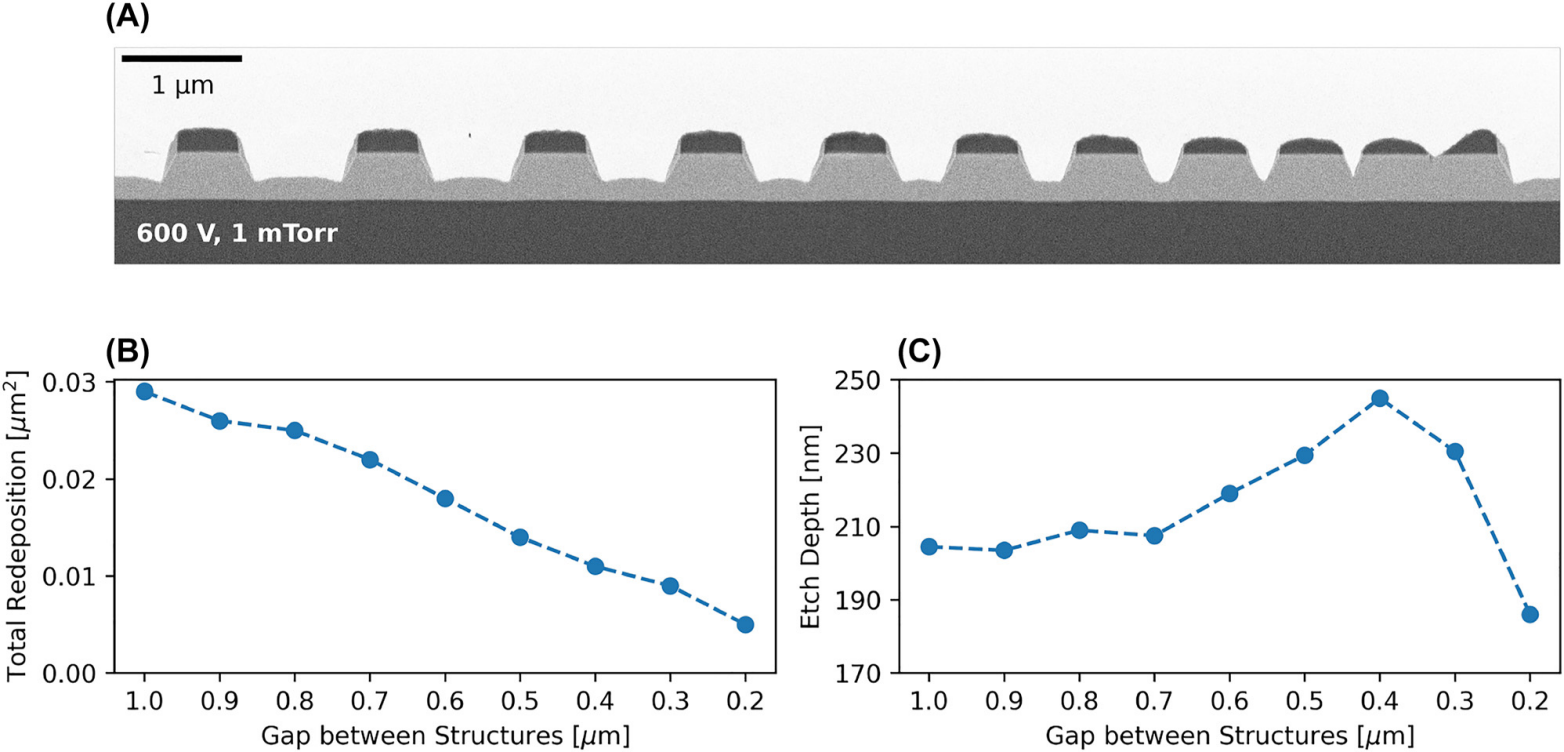
Aspect ratio dependent etching. (A) FIB cross-section of the structures of interest, constituted of an array of 500 nm-wide bars spaced by gaps of varying width, from 1 μm to 100 nm. Etched with 300 W ICP power, 600 V DC bias and a chamber pressure of 1 mTorr. (B) Total redeposition area decreasing with gap size and (C) etch depth versus gap size showing the merging of the trenches at 400 nm, as well as loading becoming evident at 300 nm. The datapoint for a gap of 100 nm is omitted.

Aspect ratio dependent etching (ARDE) is an effect which describes how a structure or film is etched depending on the ratio between exposed surface area and masked surface area. To investigate this effect in our case, we etch 200 nm into the film and analyse an array of straight lines with gradually increasing density and constant mask height. We measure the redeposition for each gap as well as the etch depth (the height of the remaining film in the center of the gap). The gap size decreases from 1 μm to 100 nm in steps of 100 nm. A FIB cross-section for such a set etched with the parameters found above is depicted in Figure 4(A), where the smallest gap of 100 nm has not opened due to the chosen ICP parameters. Figure 4(B) shows that the redeposition starts to decrease with decreasing gap size while Figure 4(C) shows an increase in effective etch depth before reverting back abruptly at a separation of 400 nm. The former is explained by ions being able to bounce between sidewalls before escaping, contributing to the etch more than once. This can also be seen in the change of shape and decrease of mask for denser areas (reduced selectivity). The change in etch depth is a combination of two effects. Firstly, the respective trenches of opposing sidewalls start to merge when the gap reduces in size (up to 400 nm) and the convex shape between sidewalls seen in Figure 4(A) starts to disappear. Secondly, we see loading for gaps below 400 nm, where ions are not able to reach the bottom with high enough energy (or reach it at all) to effectively remove material, resulting in a locally lower etch rate.

Figure 4(B) shows that a redeposition-free etch can be achieved by increasing structure density. We report that for an ICP power of 300 W, DC bias of 600 V, and chamber pressure of 11 mTorr, the dense structures are free of redeposition down to a gap of 1 μm. We argue that using a positive resist could be an option for achieving redeposition-free etching reliably. However, we cannot comment on how large the gap between waveguide and remaining slab can be before redeposition returns. Since the pressure needed here is higher than the 5 mTorr–7 mTorr reported above, single structures will and indeed are damaged and do not show a well-defined trapezoidal shape anymore (see Figure 3(D) and (E)).

### 3.4 Etch depth and IBE

**Figure 5:**
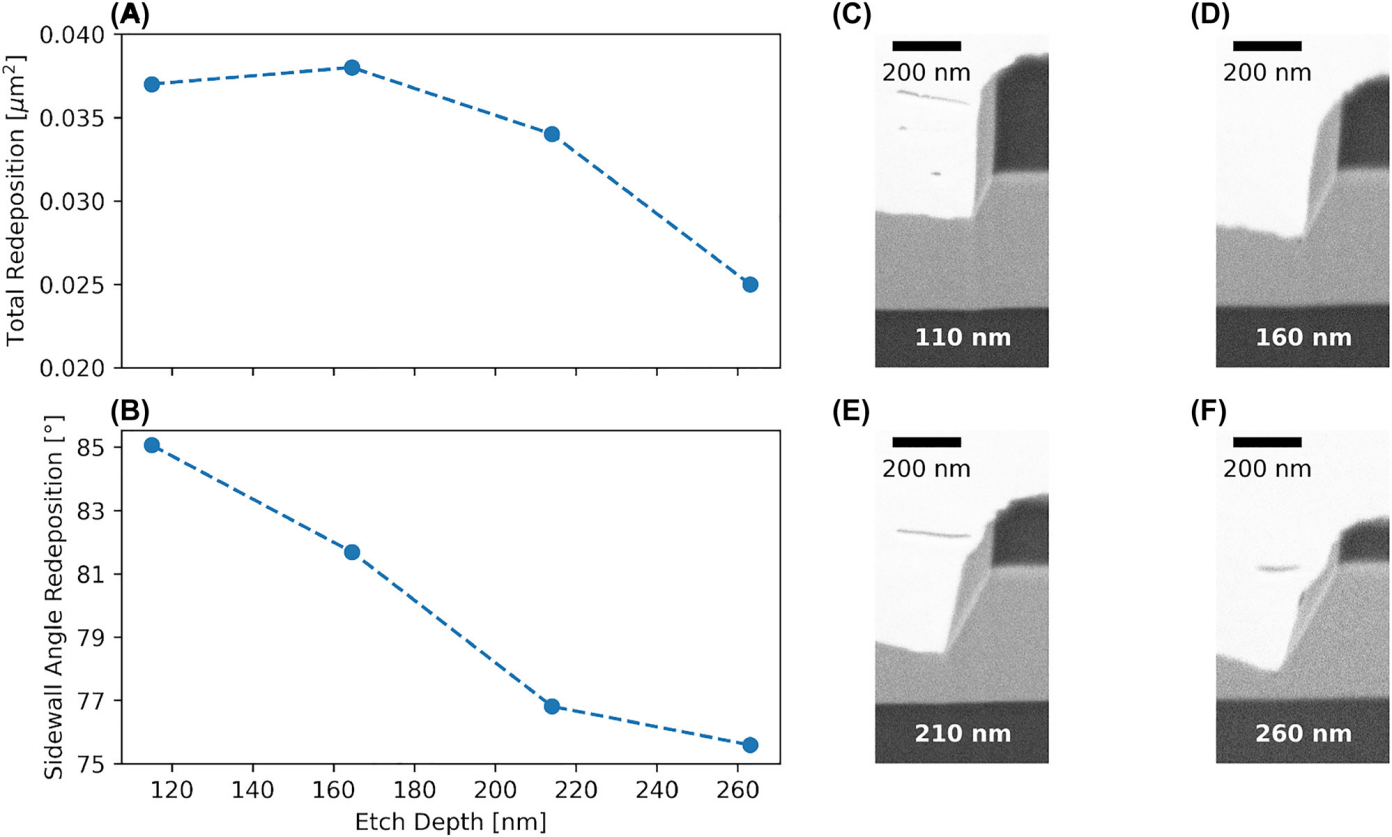
Evolution of etch depth from 110 nm to 260 nm. (A–B) Total redeposition and redeposition sidewall angle is reduced with increasing etch depth due to change in geometry and area of available surfaces to stick to. (C–F) FIB cross-sections for the different etch depths visualising the effect. ICP 300 W, Ar flow 10 sccm, pressure 1 mTorr, DC bias 600 V, resulting in an etch rate of 24 nm/min.

Figure 5 shows how a 1 μm wide structure evolves when sweeping the etch depth from 110 nm to 260 nm. The graph in (A) and the FIB cross-sections (C–F) show that the total amount of redeposition is reduced during the etching. The trench at the foot of the structure retains a trench-to-etch-depth ratio of 1:5. The redeposition first sticks to the mask at an angle of 85°. As the mask starts to get etched and more of the angled sidewall of the etched structure appears, the redeposition starts to follow the new geometry and approaches the 60° angle of the etched structure. Figure 5(C–F) shows how it changes in size and shape, gradually exposing more area of the redeposition to ions impinging from the top and increasing its effective etch rate. We argue that starting from a trapezoidal shaped mask can help in reducing redeposition as the area of attack for the impinging Ar ions is increased, while simultaneously benefiting from the polishing character of the Ar process.

**Figure 6:**
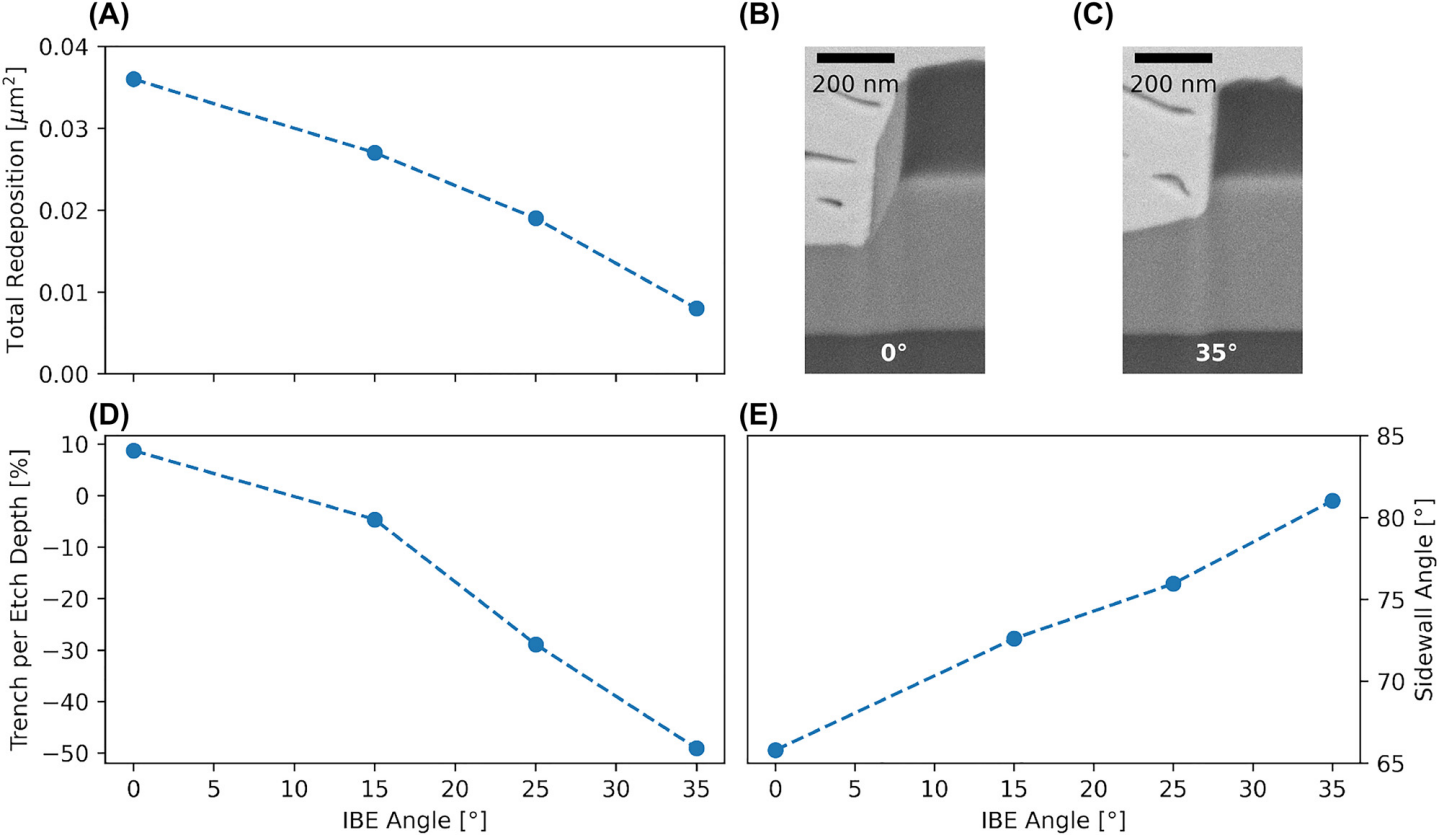
IBE results simulating a trapezoidal mask of 85°, 70°, 60°, and 50°. (A) Shows a decrease in redeposition while (E) shows an increase in resulting sidewall angle. (D) For larger angles a plateau at the foot of the structure starts to appear resulting in a negative trench. (C) Only marginal amount of redeposition left on the sidewalls. IBE parameters are: extraction current 500 mA, voltage 600 V, acceleration voltage 390 V, Ar flow 10 sccm, temperature 15 °C, revolution 20 rpm, and pressure 0.07 mTorr.

To support our argument, we etch four samples in an ion beam etcher (IBE, Ionfab 300 Plus, Oxford Instruments) at incidence angles of 0°, 15°, 25°, and 35° for the Ar ion beam. Taking into account the initial mask angle of 85°, we simulate the situation for a trapezoidal mask with sidewall angles of 85°, 70°, 60°, and 50°. Figure 6(A) shows that the amount of redeposition on the sidewall starts to decrease with increasing IBE angle, while Figure 6(E) shows an increase of sidewall angle for the structure. Figure 6(D) shows the trend of the trench where negative values visualise the formation of a plateau towards the sidewall with increasing IBE angle and is characteristic of the IBE process itself. We conclude that starting from an already trapezoidal mask helps in achieving redeposition-free etching.

### 3.5 Reproducibility and optical results

**Figure 7:**
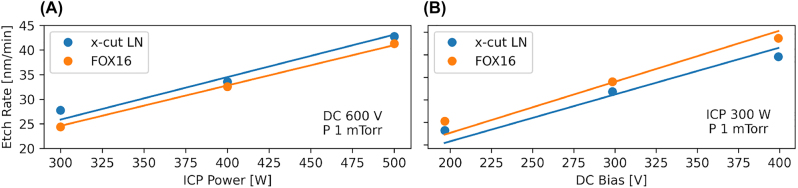
Relation of etch rate to machine parameters for fixed pressure of 1 mTorr showing the linear nature of the process. (A) Linear fit of the etch rate for fixed DC bias of 600 V with a slope of 0.09 nm/min per watt of ICP power. (B) Linear fit of the etch rate for fixed ICP power of 300 W with a slope of 0.10 nm/min per volt of DC bias.

Figure 7(A) shows the etch rate for chamber pressure of 1 mTorr and DC bias of 600 V while varying the ICP power. The slope of the data (linear fit) is 0.09 nm/min per watt of ICP power. Figure 6(B) shows the etch rate in a similar manner while varying the DC bias at the same pressure and fixed ICP power of 600 W. The slope is 0.10 nm/min per volt of DC bias. The data shown here reproduce, up to machine difference, the results found in [31] and confirm the linearity of our process.

**Figure 8:**
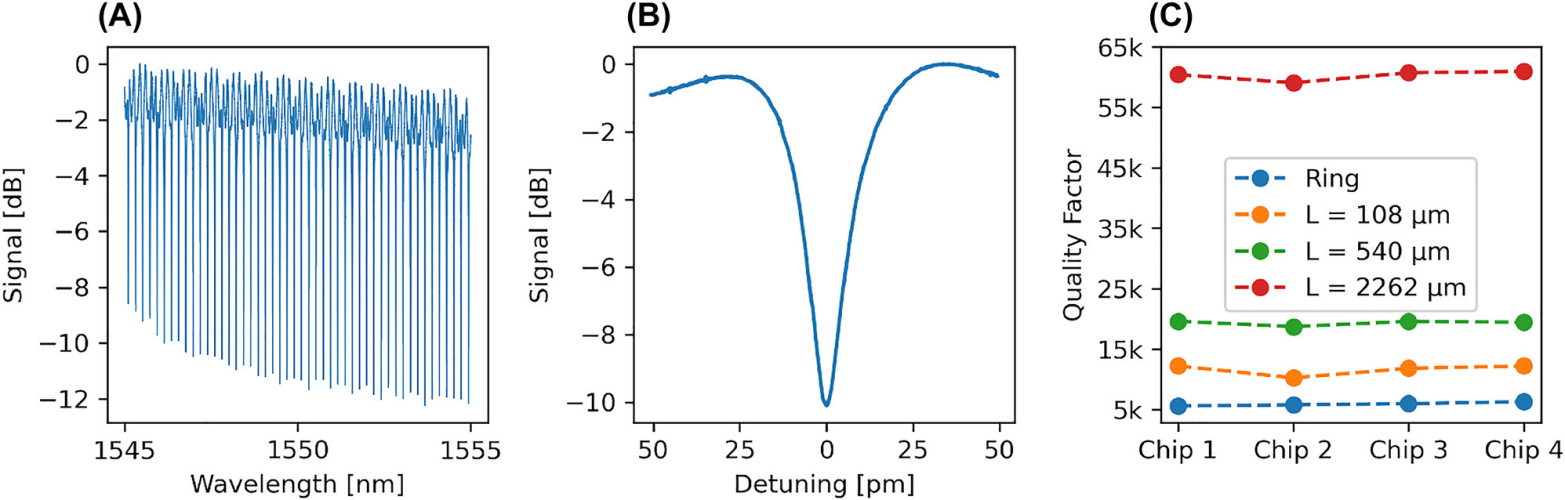
Results of the optical measurements for four repeated etches using the same parameter set. (A) Spectrum of the longest racetrack resonator on chip 4. (B) Zoom-in on the resonance closest to 1550 nm with a linewidth of 25 pm. (C) Q-factors for each device with less than 2% variation between each sample.

To test repeatability and consistency of results in terms of optical performance, we prepare four samples etched with 300 W ICP power, 600 V DC bias and chamber pressure of 1 mTorr. Although this set of parameters does not yield a redeposition free etch, it proved resistant to machine fluctuations and shows good results in terms of etch homogeneity, structure profile and optical quality. The optical properties of the etched structures are determined by measuring ring and racetrack resonators of different lengths to estimate the waveguide propagation loss [5, 10, 30, 45]. Samples are cleaned from redeposition in a 70 °C potassium hydroxide 40% solution for 30 min followed by extensive rinsing. The remaining mask is removed using a 30 s buffered hydrofluoric acid etch. No additional cladding is deposited nor are the samples thermally treated, both of which have been shown to improve optical quality significantly [3]. The waveguides have a top width of 600 nm and operate at the edge of single mode operation for the TE mode in the telecom C-band. We chose such a small width to optically probe the surface of the etched structure via the evanescent field of the mode. The bend radius is fixed at 80 μm and the length of the straight sections is 108, 540, and 2262 μm. We couple light using grating couplers with SMF28 fibers and sweep the wavelength around 1550 nm. Figure 8(A) shows a spectrum of the longest racetrack resonator and (B) is a zoom-in on the resonance closest to 1550 nm with a linewidth of 25 pm. Figure 8(C) shows the Q-factors for the resonators of the four different samples etched with a variation of less than 2% and with a calculated propagation loss of 1.55 dB/cm. The time between the etching of each sample was several days with the machine being used by different users in between. The consistent results highlight the reproducibility of our process.

In terms of influence of etch parameters on the optical quality for devices where redeposition after etching is still present, we do not observe any significant correlation besides higher quality with increasing etch depth. This is explained by stronger mode confinement due to a higher effective refractive index. Further studies are needed to give a thorough conclusion on the influence of redeposition on the optical quality.

## 4 Conclusion and outlook

We demonstrated the influence of DC bias and pressure on the redeposition formed during etching of LNOI. We found that using an appropriate set of parameters allows having a redeposition-free etch using a standard high-quality mask with nearly vertical walls. However, this comes at the cost of a very sensitive system which needs to be maintained at a high level. This result can be hard to achieve in a research environment where the machine is used for different processes and materials. These requirements can be relaxed by looking at dense structures and using a positive mask to pattern the devices. Keeping a remaining slab in proximity of the waveguide devices would improve the etching profile, however, the necessary separation between the waveguide and the slab to minimise optical loss needs further investigation. A second approach to achieve redeposition-free etching is to move away from a mask with near perfectly straight walls towards a trapezoidal shape. As this is already used to form electrical connections between different levels, a process to build upon is most likely already available in many cleanrooms. Here, a SiO_*x*_ PECVD deposition as hard mask which the lithography mask is transferred to is a promising approach. However, SiN_*x*_ or a-Si might be a better option as both can be removed without damage to the thermal oxide layer beneath the lithium niobate in case a full etch is desired. Nonetheless, this method can be sensitive to etch homogeneity of the hard mask over the full sample or wafer. Naturally, using 3D lithography or reflow of a polymer mask is also a possibility. Lastly, the influence of the ICP power and flow rate of the Ar gas on the redeposition needs further investigation.
